# The “House of Trust”. A framework for quality healthcare and leadership.

**DOI:** 10.12688/f1000research.149711.1

**Published:** 2024-05-17

**Authors:** Kris Vanhaecht, Peter Lachman, Charlotte Van der Auwera, Deborah Seys, Fien Claessens, Massimiliano Panella, Dirk De Ridder, FlaQuM research group, Ann Baeyens, Anneke Jans, Astrid Van Wilder, Brenda Droesbeke, Dirk Vanrenterghem, Els Van Zele, Emanuel Van Hoecke, Eva Marie Castro, Gerda Verheyden, Ines Van Giel, Ingrid Roosen, Jef Vanderoost, Jeroen Verhaeghe, Karolien Pennewaert, Kathleen De Sutter, Koen Vanachter, Kristin Muller, Kristof Simoens, Lieven Hoebrekx, Mieke De Medts, Nele Vanstraelen, Nele Yperman, Nina Donvil, Sofie Wijnen

**Affiliations:** 1 Leuven Institute for Healthcare Policy, KU Leuven, KU Leuven University of Leuven, Leuven, Flanders, Belgium; 2 Department of Quality, University Hospitals of Leuven, Leuven, 3000, Belgium; 3 Royal College of Physicians of Ireland, Dublin, Leinster, Ireland; 4 Department of Translational Medicine, University of Eastern Piedmont, Novara, Italy

**Keywords:** Trust, Quality, Kindness, Co-production, Values

## Abstract

In healthcare, improvement leaders have been inspired by the frameworks from industry which have been adapted into control systems and certifications to improve quality of care for people. To address the challenge to regain trust in healthcare design and delivery, we propose a conceptual framework, i.e. the “House of Trust”. This House brings together the Juran Trilogy, the emerging concept of co-production in quality management and the multidimensional definition of quality, which describes core values as an integral part of the system to deliver person- and kin-centered care.

In the “House of Trust” patients, their kin, healthcare providers, executives and managers feel at home, with a sense of belonging. If we want to build a care organization that inspires and radiates confidence to all stakeholders, highlighting the basic interactions between front- and back-office is required. An organization with both well-organized back- and front-offices can enable all to benefit from the trust each of them needs and deserves.

A quality system does not depend on government inspection and regulations nor on external accreditation to develop itself into a House of Trust. Success will only be achieved if all involved continuously question themselves about the technical dimensions of quality and their core values during the “moment of truth”.

## Introduction

Patients, kin, healthcare providers, executives and managers have high expectations on all aspects of care. To meet these expectations people must trust the healthcare service. Trust is defined as ‘ *the expectations of the public that those who serve them will perform their responsibilities in a technically proficient way, that they will assume responsibility and not inappropriately defer to others, and that they will make their patients’ welfare their highest priority’.* ^1^ The trust patients have in physicians is associated not only with patient satisfaction, but also with continuity of care, more beneficial health behaviors, less symptoms, higher quality of life and adherence to treatment. ^1^^,^^2^

Trust is so fundamental to the patient physician relationship that is it easy to assume it exists as part of the clinical interaction. This is not necessarily the case and, when trust is lost, no single approach will rebuild, maintain or ensure trust. Several key factors have been defined to increase the trust between patients, the organization and the involved teams. ^1^ These include measurement of patients’ experience, clinician engagement, a commitment to meeting the needs of patients as the core of the organizational culture, effective clinical teams, and inclusion of patients in all phases of this work. ^3^

Trust is not only important in the patient-provider relationship but is also important in the trust between the healthcare providers, managers and the healthcare organization. In a study based on 360-degrees feedback reports of 87,000 leaders, Zenger and Folkman concluded that three elements can create or re-establish trust i.e., positive relationships, consistency and good judgement or expertise. ^4^ This applies to leaders and to their complex organizations, e.g. hospitals, where the front-office of care and back-office of support meet at what is termed the “hinge” point. At the hinge point care pathways, care programs, protocols and procedures guide all stakeholders towards high quality care and excellence.

Jain noted that even before the COVID-19 pandemic trust in healthcare was declining. ^5^ The misinformation that accompanied the pandemic eroded trust even more, resulting in challenges to trust in healthcare design and delivery. Consequently, citizens, healthcare providers and managers require more trust in each other and in the organizations they visit or in which they work to co-produce required changes. ^6^

A recent paper by Bates et al. reported on the continuing high levels of harm in healthcare. This implies that to implement the changes required the leadership and change models of the past have not been as effective as they need to be. ^7^ In response to this finding, Berwick commented that safety has to be a core focus of leadership. ^8^ Mineo regarded trust to be the glue binding the leader to her/his colleagues and this provides the capacity for organizational and leadership success. ^9^ The most important success point to be gained would be high quality and safety of care.

In 2001, the Institute of Medicine defined quality in six domains, i.e., safety, effectiveness, patient-centered care, timeliness, efficiency and equity. ^10^ Since then the frameworks to improve quality and safety have been based on the theories of Deming, Juran, Feigenbaum, Crosby and others who developed quality improvement methodologies in other industries. The introduction of these improvement methodologies in healthcare has been accompanied by the introduction of control systems such as accreditation, regulation and certification. ^11^

While there has been progress in improving quality, the spread and scale up of good practice has not been at pace it needs to be. New challenges to realizing high levels of quality and safety include human resources management, the energy crisis, inflation and climate change. We contend that to achieve this goal a different approach is required. This will require coproduction and co-creation to facilitate sustainable quality. The co-creation model considers the internal and external context of an organization, co-create solutions and continuously focuses on five primary pillars. ^12^ The pillars include the Juran Trilogy of quality design and planning, quality control, and quality improvement, ^13^ with the addition of quality leadership and quality culture.

One may argue that the literature and research on leadership does not require another angle or framework. However, the declining level of trust implies that the current models of quality and leadership are not adequate to meet this new challenge. To restore trust, healthcare requires a recalibration of how we view the different components of the system, how we communicate with people in the system and how we learn to improve continually. Lee et al. (2019) have suggested a framework to improve trust in healthcare which includes concepts of leadership, measurement of trust, transparency, use of data to demonstrate trust, co-producing care with people and ensuring patients are actively engaged in care. ^3^ Another framework, the multidimensional model, includes person- and kin-centered care, resilience, transparency and leadership together with the technical quality domains and core values of kindness respect integrated care and coproduction. ^6^

In this paper, we offer a new conceptual framework that brings together the different frameworks in healthcare and place people at the center to co-produce trust and quality of care in which leadership is a shared endeavor. The *House of Trust* facilitates the implementation and further development of quality in which people (patients), their kin, healthcare providers and leaders and managers have a sense of belonging by co-creating future-proof organizations.

## The front-office, back-office and the “moment of truth”

Real care and service delivery takes place at the hinge point of the front-office and back-office of a care organization. ^14^ It is a co-production mechanism between the service user and the service provider. ^15^

• In the back-office, processes, protocols and care pathways are designed, but they come to life in the front-office. Healthcare staff and managers are trained in the theoretical models that include the latest evidence to bring their knowledge and skills to an optimal level. It is similar to the kitchen of a restaurant, where food is prepared in a seamless manner. The diner does not know how the meal is prepared and perhaps does not need to know, as there is trust that the process is hygienic, and the food will be safe to consume.
• The front-office in healthcare is where the unique meetings between the care receivers and providers happen, a real human interaction. This unique interaction takes place between a person as a patient, their loved one or kin and the individual caregiver as a person; or within the team itself, in their clinical microsystem. This is a unique moment, the *moment of truth*, which cannot be reversed if it is suboptimal. ^16^^–^^19^ The *moment of truth* requires effective bi-directional communication and education in a dynamic, authentic and at times equal partnership. The *moment of truth* includes the design of the setting, the completeness of the knowledge-gathering, and the adaptation of the persons, resources and settings to the needs of both involved in the unique relationship. ^20^

To build a care organization that inspires and radiates confidence to all stakeholders, we must highlight the interactions between the front- and back-office. We contend that an organization with well-organized back- and front-offices can enable trust and quality for patients, kin, healthcare providers, leaders and managers.

## Building a *House of Trust* to enable authentic *moments of truth*

**Figure 1.**
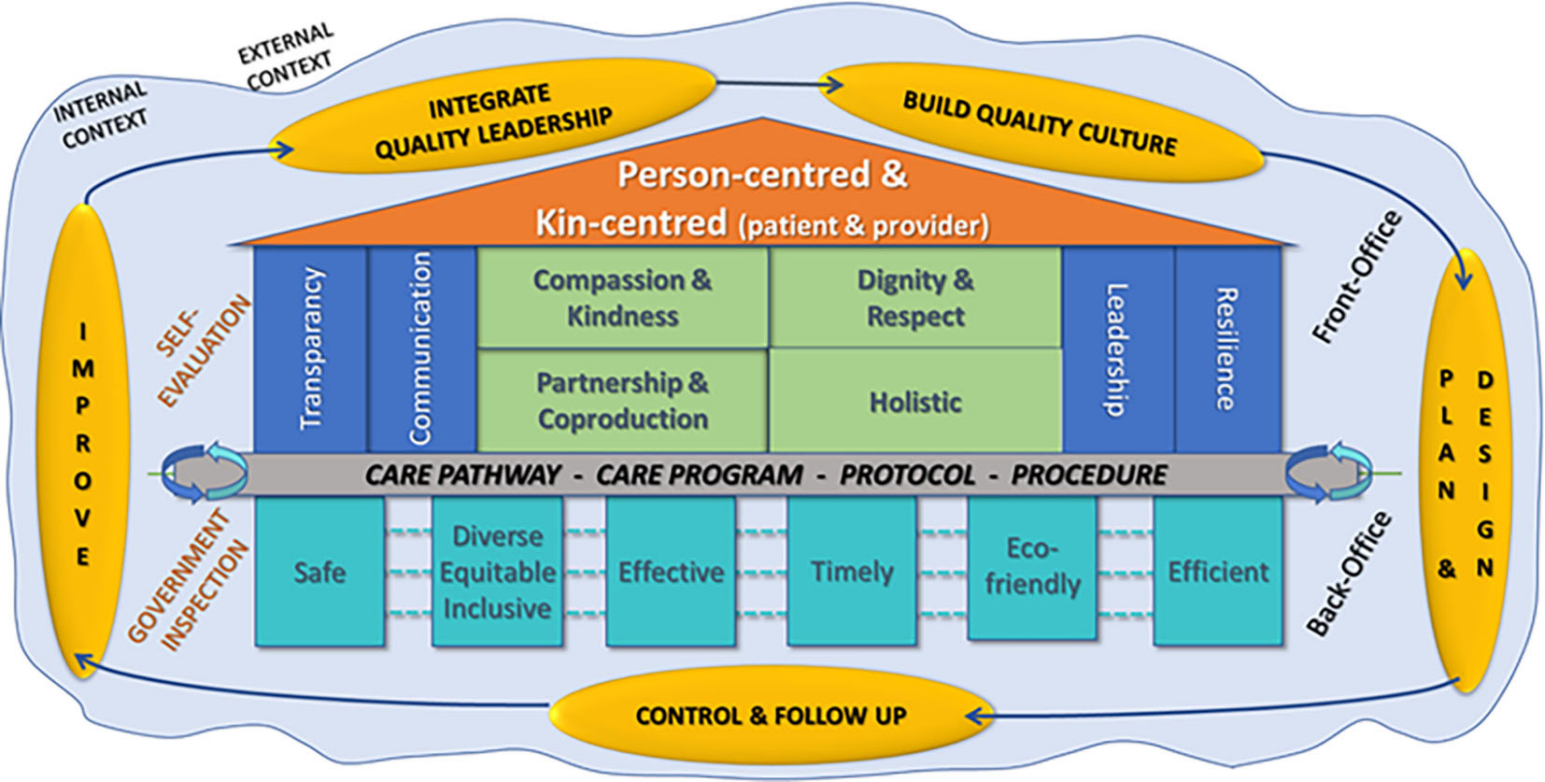
House of trust.

There are five stages to building a *House of Trust* to enable the *moment of truth* ( Figure 1).

1. **The core of the House** (green squares in the middle of the house in Figure 1):
  Care quality takes place in the front-office during the *moment of truth.* Therefore, the starting point of the multidimensional vision model is to prioritize the four core values of dignity and respect, a holistic vision, partnership and co-production and attention to compassion with kindness. ^6^ These values apply not only to the unique interaction between people in their roles as healthcare providers, patient or kin, but also between people as healthcare providers themselves or with their managers. ^17^ The core values are located in the heart of the *House of Trust*, where interaction, positive resonance, humor and acts of kindness (e.g. *Mangomoments)* can take place and people meet in-person or virtually. ^18^^–^^20^
2. **The foundations** (grey rectangle in Figure 1):
  This interaction can only be smooth, warm and of high quality if the care processes, programs, protocols and procedures are well developed and managed. ^21^ The clinical pathways and procedures are the floorboards of the House and should be developed on a solid foundation, rather than on loose sand. The foundations of a *House of Trust* are based on the technical dimensions of the multidimensional quality model and are the real hinge point between the front-office and back-office of the organization.
3. **The support posts** (turquoise squares in Figure 1):
  The domains of quality are the support posts for the *House of Trust.* Safety and efficiency serve as the outer bearing posts of the House as an unsafe or inefficient organization cannot provide quality or trust. ^22^ The other support posts are inclusive equity and diversity, effectiveness, timeliness and ecological sustainability. ^6^ The six supporting post form the backbone of a healthcare organization and are the technical dimensions of quality. These structures must be in good order, and without them, real care cannot take place.
4. **The support pillars** (blue rectangles in Figure 1):
  Four support pillars are located in the front-office and are also connected to the back-office. These pillars are transparency, communication, resilience and leadership. We need to communicate transparently, both about the unique interaction on an individual level and about our business processes which operate in the background. Transparent public reporting as well as internal openness and communication with our own stakeholders ensures that there is trust in the organization. A continued focus on clinical leadership and resilience is important to ensure that the philosophy of care is aligned across all processes. The pandemic highlighted that authentic clinical leadership, exemplary behavior and knowledge is important, as well as ensuring healthcare providers’ and managers’ physical and psychological wellbeing and resilience. ^23^ Resilience of the individual person as a patient, their kin, and the people providing care and managing the organization is essential. This, in turn, will have an impact on the attractiveness of the organization as an employer, retention of staff and creation of trust.
5. **The roof** (orange triangle in Figure 1):
  The roof is supported by the other structures and refers to continuous attention to person-centered and kin-centered care in all that the organization does. Person-centeredness is about the human experience and relationships of both the people known as patients and the people known as healthcare providers and managers, i.e., all the stakeholders in this eco-system. ^24^ A *House of Trust* can be built step by step when all structures below the roof are of high quality, and people trust each other and trust the organization. The roof can be the visiting card of the organization, which can be seen from afar to invite people to seek or provide care.

**Principles in building a House of Trust** (yellow ovals in Figure 1).

Several principles must be applied when building a *House of Trust.* Just as a real house is built brick by brick, connected to each other, with architects, surveyors and builders, so a *House of Trust is* built step by step, project by project and with a clear vision and mission. ^6^^,^^12^^,^^17^ By doing so, we can integrate implementation research with improvement methodology. ^6^ When building the House we have to consider the internal context of an organization, for example its financial status or governance challenges, and the external context, such as legislation or the impact of a pandemic.

It is crucial to involve all the stakeholders as partners in a true co-creation process from the very beginning. Each bring their own unique knowledge to the task of co-production. ^15^ This is why the ***planning and design module*** is situated in both the front- and back-offices of the organization. The operation of the front- and back-offices must be properly monitored and controlled. However, healthcare providers, leaders, managers, patients and kin should experience as little inconvenience as possible as a result of the ***control and monitoring***. The use of existing data sources will be crucial and must be implemented in the back-office as much as possible, including the development of automated control systems.

Scientific evidence is important to underpin quality. If the enhanced Juran Trilogy of Quality works well, then clinicians, management and the board will be able to use their ***quality leadership*** to build a quality culture. ^13^ When the ***quality culture*** is just and there is an innovative learning health network, it will be possible to continually take a critical look at the current design and quality level of the House, with the necessary psychological safety. ^25^ Teams must be closely involved in every ***improvement initiative*** and the voice of the patient and their kin must count. The change and implementation strategy is an essential driver of sustainable improvement.

***Governmental inspection or regulation*** may be required, even if the core, the supporting foundations and the pillars of the House are in good order and the systems and processes imposed by the government regulators are followed. However continuous ***self-evaluation*** by patients, kin, healthcare providers leaders, and managers will be key in keeping the front-office at a high level during the *moment of truth* and will challenge all to continuously enhance it.

## Concluding remarks

The *House of Trust* embodies the three cornerstones of trust described by Zenger and Folkman, ^4^ i.e., positive relationships, expertise and consistency, and addresses the challenges posed by Jain ^5^ and Lee. ^3^ Mate ^26^ highlighted the need to rebuild trust in healthcare and recommended that one has to empower people to develop a culture of Trust. The core of the House of Trust, with its four central values of care and supporting pillars of transparency, communication, leadership and resilience can deliver this urgent requirement and provides a new approach that incorporates the lessons of the past 20 years of improvement endeavors. It is key for the development of positive relationships that empowers people, i.e., patients, kin, healthcare providers, leaders and managers to co-produce trust together. Without these values relationships will not be trustworthy, or human-centered and quality and safety will not be achieved.

The evolution of an organization into a *House of Trust*, will only succeed if we continuously question the technical dimensions of quality and our core values during the *moment of truth.* The architectural design and the co-construction of a *House of Trust* are more likely if those involved are personally involved in the design, co-production, and continual review to improve its operation and assess its benefits. This includes transparency, communication, leadership and resilience and the application of the co-creation model itself. Only then will the personal orientation, for people i.e., patients, their kin and the healthcare workforce, truly radiate trust. This will result in people, the healthcare receivers and providers, remaining loyal with positive energy, engagement and commitment day after day.

## Ethical statement

Ethical and consent statement were not required.

## Data Availability

No data is associated with this article.
