# Supplementary material for: The “House of Trust”. A framework for quality healthcare and leadership
Source: F1000Res. 2024 May 17;13:503. [Version 1] doi: 10.12688/f1000research.149711.1 (PMC11179047; doi:10.12688/f1000research.149711.1)
Supplement: Peer review history

# Reviewer report: Reviewer response for version 1

**DOI:** 10.5256/f1000research.164203.r284797

**Published:** 2024-06-14

**Authors:** Eva Biringer

**Affiliations:** 1 Helse Fonna Hospital Trust, Stord, Norway

This opinion paper introduces a new conceptual framework –‘The House of Trust’– that builds on existing concepts and models in  the fields of health care quality and leadership. By introducing the framework the authors aim at regaining the ‘lost» trust in the delivery of health care.

 The paper represents a solid argument for the necessity of trust as basis of all aspects of care.  

 It is prizeworthy that the authors emphasise the values of partnership, co-production, dignity, respect etc. as core aspects in the provision of care.

 The new conceptual framework appears as very complex. It comprises a range of relevant models supported by theory and empirical findings. The framework may therefore be challenging to operationalise and implement in health care. However, it functions as argument and guiding vision for trust and trust building in and around all encounters among stakeholders in health care.

 I do not think the paper needs revision.

Is the topic of the opinion article discussed accurately in the context of the current literature?

Yes

Are arguments sufficiently supported by evidence from the published literature?

Yes

Are all factual statements correct and adequately supported by citations?

Yes

Are the conclusions drawn balanced and justified on the basis of the presented arguments?

Yes

Reviewer Expertise:

Quality improvement and care processes.

I confirm that I have read this submission and believe that I have an appropriate level of expertise to confirm that it is of an acceptable scientific standard.

# Reviewer report: Reviewer response for version 1

**DOI:** 10.5256/f1000research.164203.r284795

**Published:** 2024-06-13

**Authors:** Relinde De Koeijer

**Affiliations:** 1 Erasmus School of Health Policy & Management, Erasmus University Rotterdam, Rotterdam, The Netherlands

**Review of ‘The ‘House of Trust’: A framework for quality healthcare and leadership’**

 Dr. Relinde de Koeijer

Clarity and Accuracy of Presentation and Citation of Current Literature

- The article is well-written and clearly presents the ‘House of Trust’ framework.
- The authors cite relevant and current literature, including studies on trust in healthcare and quality improvement frameworks.
- The references are up-to-date and include key studies and frameworks that are foundational to the discussion.

Study Design and Academic Merit

- The conceptual framework is well-structured and logically presented.
- The framework addresses a gap in current models of trust and quality in healthcare by integrating different components on leadership, culture, continuous improvement, and quality of care.
- The ‘House of Trust’ framework emphasizes building trust at multiple levels. It may be interesting to reflect on how these different types of trust interact and influence each other. For example, how does trust in individual provider impacts overall organizational trust and vice versa?  Also, trust can be context dependent, it could be interesting to highlight potential specific contextual factors that could impact trust.

Methods and Analysis for Replication

- The article outlines the components and principles of the ‘House of Trust’ clearly, providing a comprehensive understanding of each element.
- It describes the interactions between the front-office and back-office and the importance of the ‘moment of truth’ in detail.

- A reflection on the ‘House of trust’ in relation to different sectors of health care could be interesting. For example, in hospitals there is generally a short-term relationship with the patient, while this is often different in long-term care. Do these differences influence the way in which trust can be build according to the authors?

Statistical Analysis and Interpretation

 The article does not present any statistical analysis as it is conceptual.

Availability of Source Data for Reproducibility

 As the paper is a conceptual proposal, it does not include source data.

Conclusions Supported by Results

- The conclusions drawn in the article are logical and follow from the presented framework and supporting literature.
- The authors effectively argue that a new approach is necessary to (re)build trust in healthcare and propose a comprehensive model that addresses current gaps.

Overall Assessment

 The article ‘The ‘House of Trust’: A framework for quality healthcare and leadership’ presents a well-structured and innovative conceptual framework. It addresses an important issue in healthcare and cites current literature effectively. The article provides a solid foundation for further research and discussion on trust and quality in healthcare.​

Is the topic of the opinion article discussed accurately in the context of the current literature?

Yes

Are arguments sufficiently supported by evidence from the published literature?

Yes

Are all factual statements correct and adequately supported by citations?

Yes

Are the conclusions drawn balanced and justified on the basis of the presented arguments?

Yes

Reviewer Expertise:

Operations Management, Quality Improvement, Healthcare, Human Resource Management

I confirm that I have read this submission and believe that I have an appropriate level of expertise to confirm that it is of an acceptable scientific standard.
